# Gluten-Free Diet Only during Pregnancy Efficiently Prevents Diabetes in NOD Mouse Offspring

**DOI:** 10.1155/2016/3047574

**Published:** 2016-08-25

**Authors:** Julie C. Antvorskov, Knud Josefsen, Martin Haupt-Jorgensen, Petra Fundova, David P. Funda, Karsten Buschard

**Affiliations:** ^1^The Bartholin Institute, Rigshospitalet, 2200 Copenhagen, Denmark; ^2^Laboratory of Specific Cellular Immunity, Institute of Microbiology ASCR, 54922 Prague, Czech Republic

## Abstract

Studies have documented that the pathogenesis of autoimmune diabetes is influenced by the intake of gluten.* Aims*. To investigate the importance of gluten exposure during pregnancy and the subsequent development of autoimmune diabetes in offspring.* Methods*. Nonobese diabetic mice were divided into 7 groups to receive combinations of gluten-free and standard diet before, during, or after pregnancy. Diabetes incidence in offspring was followed in each group (*n* = 16–27) for 310 days. Insulitis score and intestinal expression of T-cell transcription factors (RT-QPCR) were evaluated in animals from the different diet groups.* Results*. If mothers were fed a gluten-free diet only during pregnancy, the development of autoimmune diabetes in offspring was almost completely prevented with an incidence reduction from 62.5% in gluten-consuming mice to 8.3% (*p* < 0.0001) in the gluten-free group. The islets of Langerhans were less infiltrated (*p* < 0.001) and the intestinal expression of ROR*γ*t (Th17) (*p* < 0.0001) reduced in mice whose mothers were Gluten-free during pregnancy.* Conclusion*. A gluten-free diet exclusively during pregnancy efficiently prevents autoimmune diabetes development in offspring and reduces insulitis and intestinal expression of ROR*γ*t (Th17).

## 1. Introduction

Gluten exposure is an important factor in the development of type 1 diabetes (T1D) [1]. Both in nonobese diabetic (NOD) mice and in Biobreeding- (BB-) rats a gluten-free (GF) [2, 3] or hydrolyzed [4] diet lowers the incidence of the disease markedly. In humans, a GF, low carbohydrate diet was recently reported to induce remission in a newly diagnosed T1D patient [5], and improved insulin secretion has been observed after 6 months of GF diet [6]. The appearance of beta cell autoimmunity may be related to the age at introduction of cereals into the infant diet [7, 8]. Further, a high dietary content of monosaccharides increases the risk of developing T1D [9, 10].

The mechanism by which gliadin influences disease development is not known. It is likely that gut immunity is involved since we [11] and others [12, 13] have reported that gluten intake confers a proinflammatory cytokine profile in multiple regulatory T cell populations, including *γδ* T cells, in mucosal lymphoid tissues. It is likely that these changes are facilitated by or even dependent on gluten peptides being able to transverse the epithelium, which we recently demonstrated in NOD and BALB/c mice (unpublished) and which was previously reported in T1D patients [14].

It is also possible that a direct effect of gluten peptides on the beta cells can influence disease development. We have shown that a 33-mer gliadin peptide can directly close the K channel and induce insulin secretion [15]. Although it is not clear at the present time whether gliadin interacts directly with the K channel or the signal is relayed through other receptors, such as TLR4, this cellular activation might increase the risk of disease development through beta cell stress [16].

Previous experiments with GF diet in animal models of autoimmune diabetes [2, 3, 17] were all carried out in animals exposed to GF diet both during pregnancy and during the neonatal period. However, the potential of gluten to affect the diabetogenic process seems to be dependent on the time of gluten introduction, both in animals [12, 13] and in humans [7, 8]. It is therefore not known if there is a critical period, during which the effect of a GF diet is most efficient, or if both time slots are equally important. This was investigated in this study, which showed that GF diet, exclusively during pregnancy, far exceeds the efficiency of other treatment periods.

## 2. Methods

### 2.1. Animals

**Figure 1:**
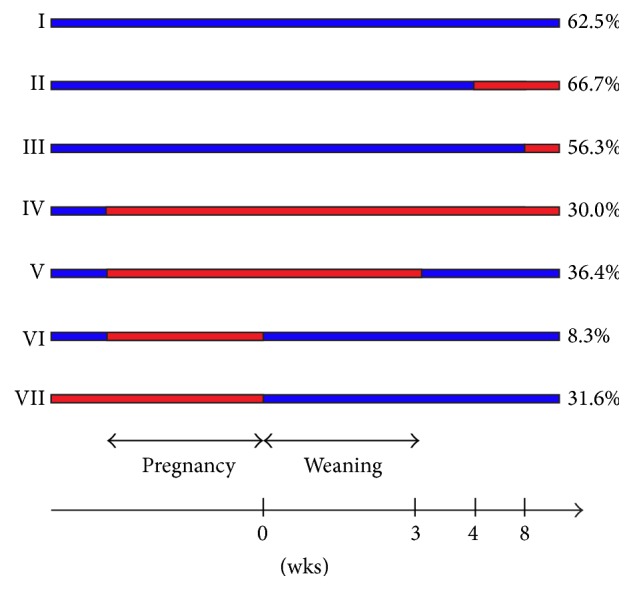
Experimental design of the seven groups of animals (breeding pairs and subsequent offspring). Administration of GF diet is shown in red and that of normal, gluten-containing chow is shown in blue. In groups IV–VI, the GF diet was initiated at the time of mating.

Breeding pairs of NOD mice were purchased from Taconic US, Taconic Europe A/S, Ry, Denmark, arrived at 4 weeks of age, and were divided into 7 groups receiving GF diet at different time points (Figure 1). Mating was initiated when animals were 7 weeks old. Pregnancy (21 days) and weaning period (21 days) were carefully recorded to allow precise timing for diet changes. After weaning, the incidence of type 1 diabetes was monitored among female offspring. The groups were as follows: Group I, breeding pairs and offsprings received STD diet throughout the study; Group II, breeding pairs were on a STD diet and offspring received GF diet from 4 weeks of age; Group III, breeding pairs were on a STD diet and offspring received GF diet from 8 weeks of age; Group IV, breeding pairs and offsprings received GF diet from mating time, during pregnancy, and throughout the study; Group V, breeding pairs received GF diet from mating time and during pregnancy and weaning and offspring was then put on a STD diet; Group VI, breeding pairs were put on a GF diet from mating time and during pregnancy and after delivery on a STD diet and offsprings continued the STD diet; and Group VII, breeding pairs were on a GF diet from arrival, mating, and pregnancy and after delivery on a STD diet and offsprings continued the STD diet. Diabetes incidence was followed in each group of offsprings (*n* = 16–27) for 310 days.

The mice were kept in a Specific Pathogen-Free (SPF) animal facility (temperature 22 ± 2 degrees, 12 h light cycle, air change 16 times per hour, and humidity 55 ± 10%) with free access to water and food. The cages had nesting materials and a cardboard shelter. The animal experiments were carried out with approval from The Animal Experiments Inspectorate (2012-15-2934-00086), and experiments were regulated by Directive 2010/63/EU on the protection of animals used for scientific purposes.

### 2.2. Diets

The animals received either the STD, nonpurified Altromin diet or a GF, modified Altromin diet (Altromin, Lage, Germany). Both experimental diets were nutritionally adequate with a similar level of protein, amino acids, minerals, vitamins, and trace element, and only the protein source differed between the diets. The exact composition of the STD and the GF diet is given in Table 1. The protein contents of the GF diet and the STD diet were similar (22.7% versus 22.9%). Proteins in the STD diet were derived from wheat (25%), maize, and soya, whereas the GF diet protein source was meat and soya proteins. The two diets also had the same content of amino acids, minerals, vitamins, and trace elements. The contents of gliadin in the STD and GF diet (Table 1) were measured by the GlutenTox ELISA Competitive assay (Biomedal Diagnostics, Sevilla, Spain). The weight of the mice was monitored, and both groups of animals displayed similar weight distribution.

### 2.3. Diabetes Incidence

NOD females were inspected daily for diabetes and from 84 days of age screened weekly for glycemia using FreeStyle Lite (Abbott) glucose monitoring. Diabetes diagnosis was based on two positive glycemia readings >12 mM with an interval of two days. The date of the first positive glycemia reading was used as diabetes onset date. Mice were killed at diabetes onset or at the age of 310 days. If animals died from other causes, this was noted, and values were excluded when calculating incidence.

### 2.4. Insulitis Score

Five mice in each group were used to evaluate insulitis score. Pancreas was removed, fixed in 4% paraformaldehyde overnight, embedded in paraffin, and sectioned in 4 *μ*m sections that were subsequently stained in hematoxylin and eosin. The sections were evaluated randomly and blinded by microscopy. Each section was scored 0 through 4 according to the following scale: (0) intact islets, (1) peri-insulitis, (2) moderate insulitis (<50% of the islets infiltrated), (3) severe insulitis (>50% of the islets infiltrated), and (4) islet destroyed.

### 2.5. RNA Isolation

Jejunal samples were stored in RNAlater (Sigma, USA) and RNA was isolated using the TRIzol procedure (Invitrogen, USA). Tissues were homogenised with a Polytron PT10-35 (Kinematica, Switzerland) at medium speed for 10 s on ice. RNA was dissolved in sterile water and precipitated with sodium acetate. Quality and concentration were measured on a NanoDrop 1000 Spectrophotometer (Thermo Scientific, USA).

### 2.6. Real-Time Quantitative Polymerase Chain Reaction (RT-qPCR)

500 ng of RNA was reverse transcribed into cDNA using the qScript cDNA SuperMix (Quanta Biosciences, USA) according to the manufacturer's protocol. cDNA was diluted 1 : 1 with sterile water.

Quantification of specific mRNA was carried out with FastStart SYBR I qPCR mixture (Roche Diagnostics GmbH, Germany). The cell type is indicated in parenthesis that is characterized by the specific transcript. Primers, designed for an annealing temperature of 60°C using Primer3 software [18] and produced by TAG Copenhagen (Denmark), were as follows: Cyclophilin A (GTG GTC TTT GGG AAG GTG AA and TTA CAG GAC ATT GCG AGC AG), Foxp3 (Treg) (CAA TGT GGC CAG TCT GGA AT and ATG ATC TGC TTG GCA GTG CT), T-bet (TH1) (CAT GGA GAA CGG AGA ATG GA and CCG CAG TCA CCT GAG TCT TC), Gata3 (TH2) (GGA AAC TCC GTC AGG GCT AC and GTG GAT GGA CGT CTT GGA GA), ROR*γ*t (TH17) (CCG CTG AGA GGG CTT CAC and TGC AGG AGT AGG CCA CAT TAC A), gamma-delta T cells (intraepithelial lymphocytes) (CAC CAA GCT AGA GGG GTC CT and GTC CGG GCC TTC ATA AAC AT), and NKp46 (NK cells) (ATG ACA CAC CCA ACC TCT GG and TCA CTG GGA AAA GAC CAT GC). Quantification was performed on a LightCycler II (Roche, USA) with initial denaturation at 95°C for 10 min followed by 45 cycles of denaturation at 95°C for 10 s, annealing at primer-specific temperatures, that is, 56–60°C for 5 s and extension at 72°C for 15 s. Sequence-verified (GATC, Germany) PCR products were used to construct standard curves for each target, and these were applied to calculate the absolute expression levels by LightCycler Software version 4.05. Specificity was checked by melting curve analysis and unspecific reactions were treated as outliers. Results were normalized relatively to the housekeeping gene cyclophilin A.

### 2.7. Statistical Analysis

The cumulative diabetes incidence was assessed using Kaplan-Meier estimation, and log-rank test was used to calculate significant differences between the groups. RT-qPCR data from groups with small sample size were log-transformed and the statistical significance of differences was analysed by one-way ANOVA followed by Bonferroni's multiple comparison test. RT-qPCR data from groups with sufficient sample size were analysed by Student's unpaired *t*-test. Data are presented as mean ± standard deviation (SD). Statistical significance (*p* < 0.05 or *p* < 0.01) is indicated by one or two asterisks in the figures, respectively. GraphPad Prism version 5.00 (GraphPad software, California) was used for the calculations.

## 3. Results

In previous experiments with GF diet in mice, the animals were fed the special diet during pregnancy as well as postnatally. To clarify which period would account for the resulting beneficial effect on type 1 diabetes development, we gave a GF diet to NOD mice at various time points as shown in Figure 1, ranging from standard diet throughout their life (Group I) to GF diet exclusively in utero (Group VI).

### 3.1. Offsprings Not Exposed to Gluten in Utero Have the Lowest Diabetes Incidence

**Figure 2:**
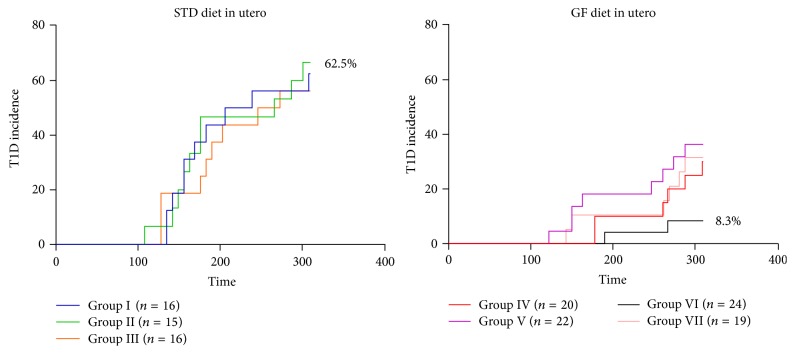
Kaplan-Meier data showing the incidence of diabetes in the experimental groups. Observation time was 310 days. The difference between groups I and VI is significant (*p* < 0.0001).

The diabetes incidence is shown in Figure 2. The incidence in the control group was 62.5% but as low as 8.3% in the group of animals that were not exposed to gluten in utero (*p* < 0.0001). Among animals GF in utero, the incidence was 30% if the mice received a GF diet after birth (Group IV), 36.4% if the diet was switched to STD diet 3 weeks after birth (Group V), and 31.6% if the mothers received a GF diet before and during but not after pregnancy (Group VII). On the other hand, if the mice received STD diet after birth and if GF diet was introduced when offspring was 4 or 8 weeks old (Groups II and III, resp.), the incidence was 66.7% and 56.3%, respectively.

### 3.2. Offspring of Mothers on GF Diet during Pregnancy Had Less Islet Infiltration

**Figure 3:**
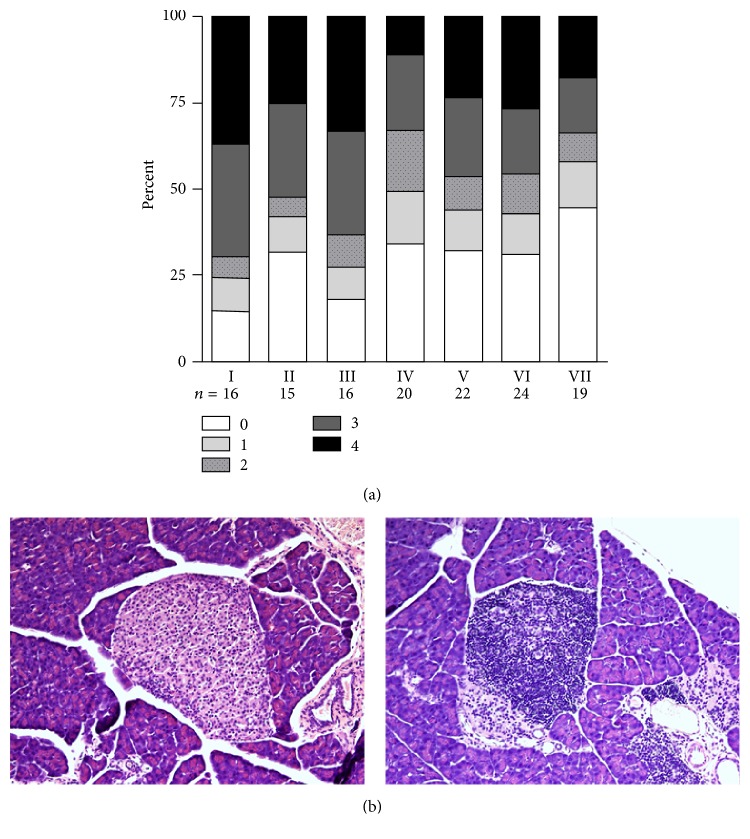
(a) Insulitis score in the experimental groups. (b) Microphotograph of normal islet (left) and severely infiltrated islet (right, corresponding to stage 3) in each group. Groups IV–VII differ from Groups I–III (*p* < 0.001).

To possibly correlate pancreatic, immunological activity with the disease incidence, we scored the islet infiltration in pancreatic sections from mice from the various experimental groups (Figure 3). In general, there was less infiltration in sections from mice that had not been exposed to gluten in fetal life than from pubs of mice that received a gluten-containing diet, but not a direct correlation. For instance, there is less infiltration in sections from Group II compared to Group I, although the incidence is almost similar. Also, the infiltration in Group VII seems less, but this is not reflected in the incidence. Over all, though, there is less infiltration in sections from mice not exposed to gluten in utero (Groups IV–VII) compared to mice exposed to gluten in utero (Groups I–III) (*p* < 0.001).

### 3.3. GF Diet in Utero Reduces the Level of Intestinal ROR*γ*t mRNA Expression in Offsprings

**Figure 4:**
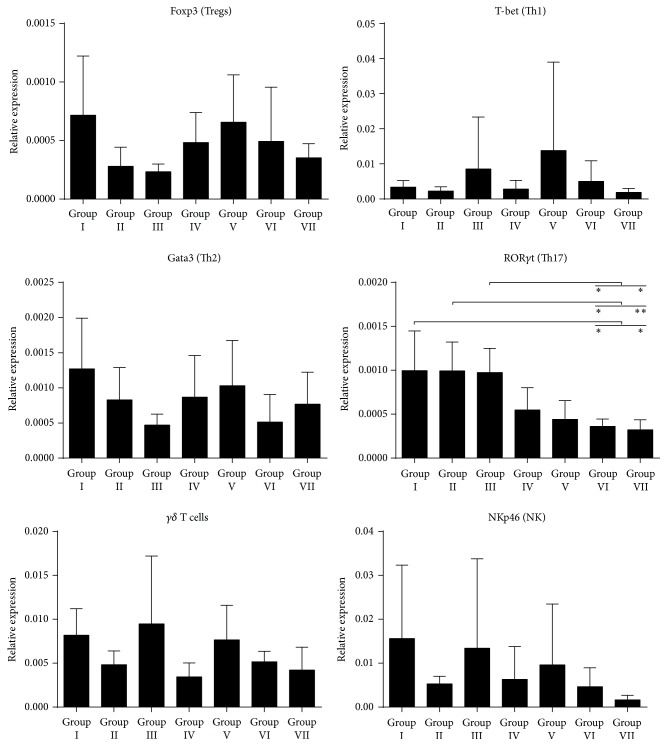
mRNA analysis of transcription factors from the jejunum, reflecting the presence of Tregs, Th1, Th2, TH17, and *γδ* T and NK cells.  ^*∗*^ denotes *p* < 0.05 and  ^*∗∗*^ denotes *p* < 0.01.

Since lymphocytes homing to the local, pancreatic lymph nodes also home to the intestine, changes in the immunological profile in the gut are likely to reflect changes in the pancreas. We performed qRT-PCR on a number of different T cell transcription factors on intestinal tissues from offspring (13-week-old NOD mice) from each of the different diet groups. Expression levels in intestinal tissue were markedly unaltered by diet (Figure 4). We did not find any diet-induced changes in the expression of Foxp3, T-bet, GATA-3, *γδ*, or NKp46. However, the expression of ROR*γ*t, a nuclear transcription factor characteristic for Th17 cells, was significantly changed by diet. When comparing mice exposed to gluten in utero (Groups I–III) with mice not exposed to gluten in utero (Groups IV–VII) we found a significant reduced expression level (*p* < 0.0001) of ROR*γ*t in the GF groups. Also, this was the only factor that consistently had lower expression for these animals compared to animals that had experienced gluten in utero. Other markers as T-bet and Gata3 (markers for Th1 and Th2 differentiation) and *γδ* T cells, characteristic for intraepidermal lymphocytes, did not differ. Also, markers for regulatory T cells (FoxP3) and NK cells (NKp46) did not differ between the diets.

## 4. Discussion

We found a remarkably low incidence of diabetes in NOD mouse offspring that was GF in utero. Although we expected that the incidence of autoimmune diabetes in the offspring of NOD mice, receiving GF diet during pregnancy, would be lower than in control animals, immediate exposure to gliadin postnatally resulted in a much lower disease frequency than in offspring of mice which stayed on a GF diet.

The importance of a GF diet in utero has also been found in a recent study [17]. Although we also observed this effect in our study (any experimental group, whose mothers received a GF diet during pregnancy, had a lower disease frequency than any experimental group whose mothers received gluten in utero), the postnatal exposure to gluten in the current study further reduced the disease incidence markedly. Thus, while there is a beneficial effect of the absence of gluten during pregnancy, there seems to be an additionally beneficial effect of exposure to gluten postnatally which largely is reflected in the islet infiltration. However, in some cases (i.e., Group V and Group VI), only minor difference is seen in the islet infiltration, suggesting functional differences between the infiltrates [19].

The mechanisms underlying these observations are not fully elucidated, but results from other investigations suggest some explanations. Thus, in mice that are fed a gluten-containing diet, gluten creates a proinflammatory cytokine profile in the pancreatic lymph nodes and leads to decreased proportions of various T regulatory subsets, including *γδ* T cells, within the mucosal lymphoid compartment [20, 21]. As we recently demonstrated that gluten fragments are readily absorbed from the intestinal tract following oral administration in mice [unpublished observation], gliadin stimulation could also take place in pancreas as well as in the intestinal mucosa. At present, it is not clear how this translates into an increased islet-directed T cell activity, but antigen spreading and an increased level of cytokines in the lymph nodes could probably help activate several immune cells unspecifically.

This mechanism could explain why a GF diet during pregnancy is beneficial, since the absence of gluten at this time would leave the mice maximally reactive to gluten antigens. Postnatal gluten exposure would then result in an increased immune respons, and gluten would then not reach the islets of Langerhans. Another explanation could be that the postnatal gluten introduction could result in a beneficial immune response in the intestinal mucosa towards gluten, perhaps mediated by the formation of a regulatory immune response to gluten peptides. If gluten is only introduced after weaning (Group V) the immune system is probably too developed and already targeted towards the pancreatic islets, resulting in a higher disease incidence.

An interesting observation is that the very low disease incidence (Group VI) is not found if the mothers also receive a GF diet before the pregnancy (Group VII). A working hypothesis for explaining this could be that the absence of gluten would decrease the level of insulin in the mother [unpublished observation], which would increase the insulin sensitivity and result in lower blood glucose values. This would delay islet maturation in the fetuses and thus not expose beta cell antigens before the thymus barrier closes. In agreement with this, neonatal glucose injections reduce the incidence of the disease, probably by increasing beta cell antigen expression and tolerance induction [22]. A human analogue to this mechanism is children of diabetic mothers that experience high glucose values in utero and develop diabetes at a lower incidence compared to children of diabetic fathers [23].

The direct translation of the results into the human disease would be to recommend the exclusion of gluten during pregnancy, but the question is if it could affect the incidence of celiac disease. Earlier, epidemiological investigations have demonstrated that the timing and mode of gluten introduction are critical for the incidence of celiac disease later in life, but recent results from prospective, randomized trials in genetically predisposed individuals showed that there was no such effect when gluten was introduced at 4, 6, or 12 months of age or whether or not children were breastfed. The same was found in children that were not genetically predisposed [24]. This suggests that the risk of developing celiac disease is unrelated to gliadin exposure. Therefore it could be interesting to test the above recommendation in a prospective human trial.

## Figures and Tables

**Table 1:** Composition of diets.

	STD	GF
Crude protein (g/kg)	227.8	228.2
Meat protein (g/kg)	84.0	153.0
Wheat protein (g/kg)	68.4	0
Gliadin	7.2 g/kg	<0.075 mg/kg
Soybean protein (g/kg)	65.3	65.3
Milk protein (g/kg)	10.0	10.0
Saccharose (g/kg)	58.0	486.7
Monosaccharide (g/kg)	6.3	6.3
Disaccharide (g/kg)	104.5	506.3
Polysaccharide (g/kg)	285.8	9.4
Crude fat (g/kg)	83.5	82.7
Crude fibre (g/kg)	28.7	28.7
Crude ash (g/kg)	71.3	61.6
Glutamic acid (g/kg)	38.6	28.1
Moisture (g/kg)	91.6	44.3
Metabolizable energy (Kcal/kg)	3640.3	3843.6
